# Reviewer acknowledgement 2014

**DOI:** 10.1186/s13756-015-0046-7

**Published:** 2015-02-20

**Authors:** Andreas Voss

**Affiliations:** Radboud University Nijmegen Medical Centre and CWZ, ᅟ, ᅟ Nijmegen, Netherlands

## Abstract

*Antimicrobial Resistance and Infection Control* would like to thank the following colleagues for their assistance with peer review of manuscripts for the journal in 2014.

**Anucha Apisarnthanarak**

Thailand

**David Birnbach**

United States of America

**Martin Bootsma**

Netherlands

**Emilio Bouza**

Spain

**John Boyce**

United States of America

**Jean Carlet**

France

**Sujatha Chandrasekaran**

India

**Esmita Charani**

United Kingdom

**Hsiu-Yin Chiang**

United States of America

**Peter Collignon**

Australia

**Christopher Crank**

United States of America

**James Davis**

United States of America

**Olivier Denis**

Belgium

**Markus Dettenkofer**

Germany

**Akinwale Efunshile**

Germany

**Mario Giuffre**

Italy

**Wolfgang Heizmann**

Ghana

**Alison Holmes**

United Kingdom

**Joost Hopman**

Netherlands

**Abubakar Hoza**

Tanzania

**Waleria Hryniewicz**

Poland

**Benedikt Huttner**

Switzerland

**Evgeny Idelevich**

Germany

**Jan Kluytmans**

Netherlands

**Robin Köck**

Germany

**Axel Kramer**

Germany

**Dortet Laurent**

France

**Matthias Maiwald**

Singapore

**Shaheen Mehtar**

South Africa

**Maria Luisa Moro**

Italy

**L. Silvia Munoz-Price**

United States of America

**Dilip Nathwani**

United Kingdom

**Lindsay Nicolle**

Canada

**Jonathan Otter**

United Kingdom

**Eli Perencevich**

United States of America

**Didier Pittet**

Switzerland

**Yok-Ai Que**

Switzerland

**Thomas Riley**

Australia

**Jesús Rodríguez-Baño**

Spain

**Cassandra Salgado**

United States of America

**Ben Semmekrot**

Netherlands

**Tesfaye Sisay Tessema**

Ethiopia

**Nuntra Suwantarat**

United States of America

**Paul Tambyah**

Singapore

**Judith Tanner**

United Kingdom

**Annet Troelstra**

Netherlands

**Christina MJE Vandenbroucke-Grauls**

Netherlands

**Jacobien Veenemans**

Netherlands

**Cees Verduin**

Netherlands

**Erwin Verkade**

Netherlands

**Margreet Vos**

Netherlands

**Maja Weisser**

Switzerland

**Andreas Widmer**

Switzerland

**Mireille Wulf**

Netherlands

